# A Machine Learning Approach Utilizing Normal‐appearing white matter Amyloid uptake for Amyloid Positivity Classification and Clinical progression prediction

**DOI:** 10.1002/alz.088639

**Published:** 2025-01-09

**Authors:** Sungman Jo, Ji Won Han, Ki Woong Kim

**Affiliations:** ^1^ Seoul National University, Seoul Korea, Republic of (South); ^2^ Seoul National University Bundang Hospital, Seongnam Korea, Republic of (South); ^3^ Seoul National University College of Natural Science, Seoul Korea, Republic of (South)

## Abstract

**Background:**

Amyloid positron emission tomography (PET) positivity is usually determined using visual assessment or a cutoff for standardized uptake value ratio (SUVR) based on cortical amyloid uptake. Our study aimed to develop and validate a machine‐learning based method for classifying amyloid PET positivity utilizing amyloid uptake in cerebral normal‐appearing white matter (NAWM).

**Method:**

We analyzed Florbetaben (FBB) PET and MRI data from 813 participants aged 50 and over, recruited from dementia clinics of Seoul National University Bundang Hospital and the Korean Longitudinal Study on Cognitive Aging and Dementia cohort. Using global cortical composite SUVR and the NAWM Dominance Ratio (NDR; ratio of global SUVR to NAWM SUVR), we developed a machine‐learning algorithm called Gaussian Mixture Model NDR (GMM NDR). This algorithm iterates through two mixture components to categorize individuals as amyloid‐positive (A+) or negative (A‐). We compared the classification performance of GMM NDR to a conventional SUVR cutoff (0.96) using confusion matrices, receiver operating characteristic (ROC) analysis, and visual assessment as the gold standard. Additionally, we explored which classification method, among the three, best predicted clinical progression in individuals with discordant amyloid positivity across methods.

**Results:**

The GMM NDR method demonstrated accuracy, sensitivity, specificity, and Youden’s index of 0.936, 0.988, 0.897, and 0.885, respectively, while the global SUVR method reported similar values of 0.941, 0.974, 0.916, and 0.890 for these metrics. The area under the curve (AUC) for each method were found to be comparable (0.943 for GMM NDR, 0.945 for global SUVR, p = 0.818). Among 59 participants with discrepancies in amyloid positivity across three methods, only the GMM NDR‐identified A+ group showed more clinical progression than the A‐ group (71.7% vs. 33.3%, p = 0.02). Logistic regression revealed a significant association between GMM NDR positivity and clinical progression (OR = 11.42, 95% CI = 1.105 – 117.92, p = 0.041), not observed in other methods.

**Conclusion:**

The GMM NDR method demonstrates both robust classification accuracy for amyloid positivity and superior ability to predict clinical progression compared to traditional methods. This suggests its potential as a sensitive marker for monitoring disease progression and informing treatment decisions in neurodegenerative diseases.

